# Long-term outcomes of patients with *Streptococcus suis* infection in Viet Nam: A case-control study

**DOI:** 10.1016/j.jinf.2017.09.019

**Published:** 2018-02

**Authors:** Vu T.L. Huong, Hoang B. Long, Nguyen V. Kinh, Ta T.D. Ngan, Vu T.V. Dung, Behzad Nadjm, H. Rogier van Doorn, Ngo T. Hoa, Peter Horby, Heiman F.L. Wertheim

**Affiliations:** a Wellcome Trust Major Overseas Programme – Oxford University Clinical Research Unit, 78 Giai Phong, Dong Da, Ha Noi, Viet Nam; b Nuffield Department of Medicine, University of Oxford, Oxford OX1 3BD, UK; c National Hospital for Tropical Diseases, 78 Giai Phong, Dong Da, Hanoi, Viet Nam; d Wellcome Trust Major Overseas Programme – Oxford University Clinical Research Unit, 764 Vo Van Kiet, Ho Chi Minh, Viet Nam; e Department of Biology and Biotechnology, University of Science -National University, Ho Chi Minh, Viet Nam; f Radboud UMC, Geert Grooteplein Zuid 10, 6525 GA Nijmegen, The Netherlands

**Keywords:** *Streptococcus suis*, Hearing loss, Vestibular dysfunction, Bacterial meningitis, Long-term outcomes

## Abstract

**Objectives:**

*Streptococcus suis* is a zoonotic cause of severe meningitis and sepsis in humans. We aimed to assess the long-term outcomes in patients who survived *S. suis* infection, in particular the progress and impact of vestibulocochlear sequelae.

**Methods:**

This case-control study evaluated outcomes of *S. suis* infection at discharge and 3 and 9 months post-discharge for 47 prospectively enrolled cases and at 11–34 months for 31 retrospectively enrolled cases. Outcomes in patients were compared to 270 controls matched for age, sex and residency.

**Results:**

The prevalence ratio (PR) of moderate-to-complete hearing loss was 5.0(95%CI 3.6–7.1) in cases at discharge, 3.7(2.5–5.4) at 3 months, 3.2(2.2–4.7) at 9 months, and 3.1(2.1–4.4) in retrospective cases compared to controls. Hearing improvement occurred mostly within the first 3 months with a change in hearing level of 11.1%(95%CI 7.0–15.1%) compared to discharge. The PR of vestibular dysfunction was 2.4(95%CI 1.7–3.3) at discharge, 2.2(1.4–3.1) at 3 months, 1.8(1.1–2.5) at 9 months, and 1.8(1.1–2.6) for retrospective cases compared to controls. Cases also indicated more problems with mobility, self-care and usual activities.

**Conclusions:**

Both hearing and vestibular impairment were common and persist in cases. Appropriate patient management strategies are needed to reduce the incidence and impact of these sequelae.

## Hightlights

• Severe hearing and vestibular impairment persists in many *S. suis* survivors.
• Hearing function tends to only improve in the first 3 months post discharge.
• Vestibular dysfunction shows little recovery during the follow-up time period.
• Survivors reported significantly lower health status and quality of life.
• Appropriate patient management strategies are needed to reduce disease impact.

## Introduction

*Streptococcus suis* is a commensal bacterium in the respiratory, genital and digestive tract of pigs, which can infect humans through penetrating injuries or consumption.^1^ *S. suis* infection is common in East and Southeast Asia.^2^ In Vietnam it is the most frequently diagnosed bacterium in adult bacterial meningitis.^3^, ^4^ The two most common presentations are purulent meningitis and severe sepsis, with case-fatality rates ranging from 0 to 56% depending on the clinical syndrome. Hearing loss and balance problems are the most frequently reported sequelae.^2^, ^3^, ^4^

Despite the growing literature on the epidemiological, microbiological and clinical aspects of *S. suis* infection, less is understood about the frequency, persistence and impact of clinical sequelae. Reported rates of hearing loss vary greatly between studies, from 6% to 100%, with the weighted pooled estimate at 39% in a recent review and meta-analysis.^2^ For vestibular dysfunction, the weighted pooled estimate is 23%, ranging from 3% to 60% of cases. Only one study has systematically followed cases of *S. suis* meningitis prospectively to quantify the prevalence of sequelae. This clinical trial of adjuvant dexamethasone found 26/46 (56%) of cases with hearing loss in the placebo group and 15/41 (37%) in the intervention group at 6 months, but did not report on vestibular dysfunction.^4^ Here, we aim to evaluate the long-term health outcomes in patients with *S. suis* infection.

## Materials and methods

### Study design and participants

This case-control study includes prospectively and retrospectively enrolled patients, and controls. We followed up prospectively enrolled patients admitted between November 2014 and October 2015 with laboratory confirmed *S. suis* infection at the National Hospital for Tropical Diseases (NHTD) in Hanoi, Viet Nam. Patients were assessed at discharge and after 3 and 9 months since discharge. To assess the long-term health outcomes beyond 9 months we retrospectively enrolled patients admitted to NHTD between January 2013 and October 2014 with laboratory confirmed *S. suis* infection who were assessed once using the same assessment tools.

Four non-patient visitors at NHTD were matched for sex, age (±5 years) and location (rural/ urban) with cases recruited between November 2014 and August 2016 and enrolled as controls. Exclusion criteria included a history of *S. suis* infection or meningitis. We assumed that these controls represent the source population from which the cases were derived, and that, except for the studied exposure, both patients and controls were similar in terms of the baseline risk of hearing and vestibular dysfunction. All cases and controls gave written consent to participate in the study. The study was approved by the institutional review board of NHTD and the University of Oxford Tropical Research Ethics Committee (OxTREC).

### Data collection

Baseline characteristics were collected from the hospital charts (cases) and through interviews (both cases and controls). Main outcomes including hearing, balance, history of vestibular symptoms, impact of hearing impairment and dizziness, visual acuity, cognitive function and overall health status were assessed for all cases and controls as described below (more details in Supplementary Material).

Air conduction audiometry was conducted according to a standardized protocol by trained staff in a sound-proof booth (for patients who visited the hospital and controls) or in a quiet place (for patients who were visited at home), following a modified Hughson-Westlake automated procedure (Audiometer AD226, Interacoustics, Eden Prarie, MN). The hearing threshold in each ear was tested for frequencies from 0.5 to 8 kHz with the first threshold tested at 1 kHz. Pure tone average (PTA) is defined as the average of hearing thresholds measured over four frequencies 0.5, 1, 2 and 4 kHz in the better ear. Impairment severities are categorized as normal (PTA < 20 dB), mild (20–34 dB), moderate (35–49 dB), moderately severe (50–64 dB), severe (65–79 dB), profound (80–94 dB), and complete (≥95 dB),^5^ and further collapsed to simplify analyses.

The Modified Clinical Test of Sensory Interaction and Balance (m-CTSIB) was used to assess vestibular function.^6^ This assesses the individual's ability to stand unassisted with feet together and arms folded across the waist with both hands holding the elbows, under 4 successive test conditions: (1) eyes open on firm surface, (2) eyes closed on firm surface, (3) eyes open on foam surface and (4) eyes closed on foam surface. Balance disorder was determined as any failure to complete the test under any test condition, and vestibular dysfunction was determined as a failure to pass test condition 4 only.

The Vertigo Symptoms Scale (VSS) was used to assess the subjects' recall of experiencing 34 items in the previous 12 months, a score between 0 (never experienced) to 4 (quite often) is given to each symptom.^7^ Total VSS scores were computed. We also specifically examined three symptoms included in this scale: vertigo (a feeling that things are spinning or moving around), dizziness (a feeling of being light-headed, “swimmy” or giddy), and unsteadiness (feeling unsteady, about to lose balance).

We used the Hearing Handicap Inventory for Adults (HHIA)^8^ and Dizziness Handicap Inventory (DHI)^9^ to assess the perceived emotional, social and physical handicap caused by hearing loss and dizziness. Visual acuity was tested using a Snellen chart. We used Mini Mental State Examination^a^ (MMSE)^10^ to evaluate subjects' cognitive function. Overall health status was assessed using the EQ-5D scale,^11^ which asked subjects to report if they had any problem on the present day for each of 5 domains: mobility, self-care, usual activities, pain/discomfort, and anxiety/depression. VSS, HHIA, DHI, and EQ-5D were only done at 3 and 9 months for the prospective patients.

### Sample size calculation

We compared the prevalence of disabling outcomes among cases and controls, and the change in the main outcomes over time. We determined the minimum sample size to detect a difference of 25% in the prevalence of vestibular dysfunction between cases and controls (assuming a similar rate of 25% among Vietnamese controls as reported among adults aged 40–60 years in the U.S.).^12^ With a sample size of 46 cases per group, the study would have a power of 90% with a 2-sided significance level of 5% to detect such a difference in vestibular dysfunction at the 1:4 case to control ratio.^13^ This sample size is also sufficient to examine the difference in the prevalence of hearing loss between cases and controls (a minimum of 21 cases per group is required, assuming a prevalence of 48% among cases^4^ and 14% among controls based on recent estimates for the general adult population in the Asia Pacific region).^5^

### Statistical analysis

To compare outcomes between cases and controls, we used log-binomial models and calculated adjusted prevalence ratios (PR), the ratio between the probability of an outcome for cases and for controls after considering potential confounders.^14^ We examined possible confounding effects of all baseline characteristics by adding each separately to the core model of exposure (disease status) and outcome.

To evaluate the change in hearing thresholds among cases over time, we excluded patients showing no response to sound stimuli at the four frequencies used to calculate the PTA and ran a linear mixed model controlling for between-patient variations (random effect) over the main effect of interest (assessment time-points) using the *nlme* package.^15^ Hearing threshold was included in the model in a log scale to improve data normality. Missing data on hearing threshold for patients who did not have audiometry were omitted in this analysis. We also evaluated if the location of audiometry (in the sound-proof booth at the hospital versus at patient's home) affect the results on hearing impairment. All statistical analyses were done with R software (version 3.2.2).^16^

## Results

**Figure 1:**
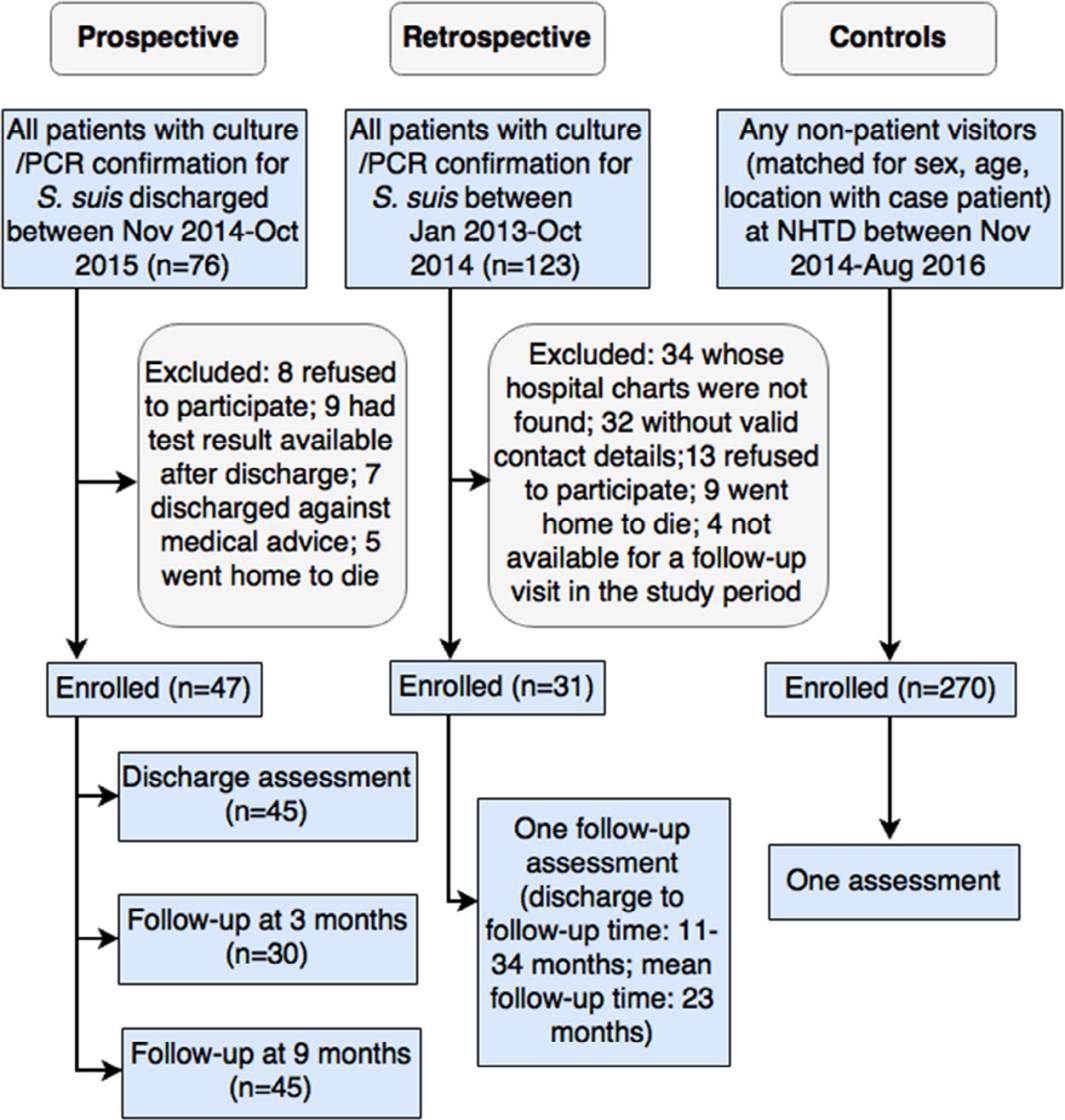
Diagram of case and control recruitment and assessment

From 76 patients admitted with laboratory-confirmed *S. suis* infection at NHTD between November 2014 and October 2015, 47 were enrolled as cases prospectively (Fig. 1). Thirty cases returned to the hospital for assessment at 3 months post-discharge. At 9 months, follow-up assessments were performed for 27 cases at the hospital and 18 at their home (for those unable to come to the hospital). An additional 31 patients were enrolled as cases retrospectively, and a total of 270 controls were recruited. Retrospectively enrolled cases were significantly older than controls since few controls of age 60 or above could be recruited from the visitors at the hospital (Table 1). Almost all (46/47 and 30/31) patients in the case groups were diagnosed with bacterial meningitis; the two remaining patients were diagnosed with sepsis syndrome.

**Table 1:** Characteristics of 78 cases (47 prospective and 31 retrospective) and 270 controls

Characteristics	Prospective cases, n(%)	Retrospective cases, n(%)	Controls, n(%)
Male sex	38 (80.8)	28 (90.3)	232 (85.9)
Age a	51.3 (12.1)	55 (12.6)^b^	48.7 (10.5)
Living in rural area	37 (78.7)	23 (74.2)	208 (77.0)
Occupation (farmer/ manual)	32 (68.1)	20 (64.5)	147 (54.4)
Education (schooling years) a	7.8 (3.1)^b^	8.9 (3.6)	9.0 (3.8)
Pre-existing medical conditions c	10 (21.3)	3 (9.7)	44 (16.3)
Clinical syndrome			
Meningitis	46 (97.9)	30 (96.8)	na
Sepsis	1 (2.1)	1 (3.2)	na
Corticosteroid treatment during admission^d^	30 (63.8)	17 (54.8)	na

Data are shown as n (%) unless otherwise stated. No significant difference was found between each of the case groups compared to the control group unless otherwise stated. na, not assessed.

a Mean(standard deviation).

b Significantly different compared to controls (t-test for continuous or chi-square test for binary variable, p < 0.05).

c Include one or more of the following conditions: arthritis, diabetes, hepatitis/ cirrhosis, renal disease, hypertension, heart disease, chronic lung disease, seizure, brain injury, paralysis, splenectomy, current steroids treatment, depression, HIV, malignancy, or any other immunosuppressed conditions.

d Corticosteroid was not used in the two sepsis patients.

### Hearing outcome

Profound-to-complete and moderate-to-severe hearing impairments were found in 14/44 (32%) and 23/44 (52%) of patients at discharge; this proportion fell to 13/45 (29%) and 13/45 (29%) at 9 months, respectively (Table 2). Impairment occurred at all measured frequencies, however, more severe loss was found in the high-frequency range. Cases had a fivefold higher proportion of hearing loss at discharge (PR5.0 for moderate or worse impairment, 95%CI 3.6–7.1 adjusted for age) (Table 3). This ratio decreased after 3 and 9 months, indicating recovery of hearing function post-discharge (PR3.7 at 3 months and 3.2 at 9 months). A similar ratio was observed among retrospectively enrolled cases (PR3.1).

**Table 2:** Description of health outcomes in prospectively enrolled cases at discharge, 3 months, and 9 months, retrospectively enrolled cases and controls

Outcome	Prospective cases, n(%)			Retrospective cases, n(%) (n = 31)	Controls n(%) (n = 270)
	Discharge (n = 45)	3 months (n = 30)	9 months (n = 45)		
*Hearing impairment*^a^					
No impairment	2 (4.5)	3 (10)	1 (2.2)	2 (6.5)	66 (24.4)
Mild	5 (11.4)	7 (23.3)	18 (40.0)	9 (29.0)	163 (60.4)
Moderate	13 (29.5)	5 (16.7)	8 (17.8)	6 (19.4)	35 (13.0)
Moderately severe	7 (15.9)	2 (6.7)	2 (4.4)	4 (12.9)	2 (0.7)
Severe	3 (6.8)	2 (6.7)	3 (6.7)	2 (6.5)	3 (1.1)
Profound	3 (6.8)	0 (0)	2 (4.4)	2 (6.5)	0 (0)
Complete	11 (25.0)	11 (36.7)	11 (24.4)	6 (19.4)	1 (0.4)
*m-CTSIB result*					
Balance disorder	37 (82.2)	20 (66.7)	29 (64.4)	20 (64.5)	79 (29.3)
Vestibular dysfunction b	16 (66.7)	15 (60.0)	16 (50.0)	13 (54.2)	75 (28.2)
*VSS symptoms*					
Vertigo	na	12 (40.0)	10 (22.2)	12 (38.7)	55 (20.4)
Dizziness	na	20 (66.7)	22 (48.9)	18 (58.1)	63 (23.3)
Unsteadiness	na	16 (53.3)	18 (40)	15 (48.4)	25 (9.3)
At least one symptom c	na	22 (73.3)	23 (51.1)	21 (67.7)	99 (36.7)
All 3 symptoms c	na	9 (30)	9 (20)	9 (29)	12 (4.4)
Visual impairment d	5 (11.1)	4 (13.3)	7 (15.5)	2 (6.5)	15 (5.5)
Cognitive impairment e	16 (40)	5 (19.2)	10 (23.8)	8 (25.8)	59 (21.9)
Hearing handicap f	na	14 (58.3)	16 (39)	11 (42.3)	16 (7.8)
Dizziness handicap g	na	10 (52.6)	10 (50)	12 (70.5)	16 (26.2)
*Having problem in each EQ-5D domain*					
Mobility	na	8 (26.7)	8 (17.8)	6 (19.4)	5 (1.9)
Self-care	na	4 (13.3)	6 (13.3)	1 (3.2)	1 (0.4)
Usual activities	na	6 (20.0)	7 (15.6)	2 (6.5)	1 (0.4)
Pain/ Discomfort	na	11 (36.7)	12 (26.7)	10 (32.3)	50 (18.5)
Anxiety/ Depression	na	10 (33.3)	10 (22.2)	2 (6.5)	132 (48.9)
Self-rated health h, median (IQR)	na	90 (70–97)	90 (70–90)	75 (60–85)	88 (75–95)

Data are presented as count (%) or otherwise specified. Two sepsis patients only had very mild hearing loss (PTA = 21.2 dB for the prospectively enrolled case at 9 months and 22.5 dB for the retrospectively enrolled case), and no balance disorder. m-CTSIB, modified Clinical Test of Sensory Interaction and Balance; VSS, Vertigo Symptoms Scale; na, not assessed; IQR, Inter-quartile range.

a At 9 months, audiometry was performed in the sound-proof booth for 27 cases, and at their home for 18 cases. 17/27 cases evaluated in the sound-proof booth versus 9/18 evaluated at home had moderate hearing impairment or worse.

b Denominator consists of those participating in condition 4 only.

c In three symptoms: vertigo, dizziness, unsteadiness.

d Defined as visual acuity in the better eye <6/18.

e MMSE score ≤ 23.

f Hearing Handicap Inventory for Adults score ≥18 (denominator consists of those with hearing impairment only).

g Dizziness Handicap Inventory score ≥ 16 (denominator consists of those reporting dizziness only).

h Subjects were asked to rate their health at the present day on a visual analogue scale, ranging from 0 (worst imaginable) to 100 (best imaginable health state) on the EQ-5D questionnaire.

**Table 3:** Prevalence ratios of main health outcomes in prospectively enrolled cases at discharge, 3 months, and 9 months, and retrospectively enrolled cases compared with the controls

Outcome	Prospective, PR (95%CI) a			Retrospective, PR (95%CI) a
	Discharge	3 months	9 months	
*Hearing impairment*^b^				
Moderate or worse	5.0 (3.6–7.1)	3.7 (2.5–5.4)	3.2 c (2.2–4.7)	3.1 (2.1–4.4)
Severe or worse	22.6 (10.5–58.5)	21.1 (9.3–56.2)	16.3 (7.2–43.2)	13.5 (5.7–37.6)
*m-CTSIB result*				
Balance disorder	2.8 (2.2–3.5)	2.3 (1.6–3.0)	2.2 (1.6–2.9)	2.1 (1.4–2.8)
Vestibular dysfunction	2.4 (1.7–3.3)	2.2 (1.4–3.1)	1.8 (1.1–2.5)	1.8 (1.1–2.6)
*VSS symptoms*				
Vertigo	na	1.9 (1.1–3.0)	1.1 (0.5–1.8)	1.7 (1.0–2.8)
Dizziness	na	2.7 (1.9–3.7)	2.0 (1.3–2.8)	2.2 (1.4–3.1)
Unsteadiness	na	5.0 (3.0–8.3)	3.9 (2.3–6.6)	3.7 (2.2–6.4)
At least one symptom d	na	2.0 (1.5–2.5)	1.4 (1.0–1.8)	1.7 (1.2–2.3)
All 3 symptoms d	na	5.8 (2.6–12.8)	3.6 (1.5–8.2)	4.0 (1.8–8.9)
*Having problem in each EQ-5D domain*				
Mobility	na	14.5 (5.1–45.7)	9.3 (3.2–29.7)	6.8 (2.1–23)
Self-care	na	64.2 (8.1–1672)	35.5 (6.2–664)	8.5 (0.3–230)
Usual activities	na	53.5 (9.5–996)	42.5 (7.8–786)	18.1 (1.8–381)
Pain/ Discomfort	na	1.9 (1.0–3.0)	1.2 (0.7–2.0)	1.5 (0.8–2.5)
Anxiety/ Depression	na	0.7 (0.4–1.1)	0.4 (0.2–0.7)	0.1 (0.02–0.4)

PR, Prevalence Ratio; CI, Confidence Interval; m-CTSIB, modified Clinical Test of Sensory Interaction and Balance; VSS, Vertigo Symptoms Scale; na, not assessed.

a Adjusted for age and also for sex whenever convergence was reached.

b Hearing loss was categorized into a binary variable using two cut-off PTAs: ≥ 35 dB (moderate impairment or worse) and ≥50 dB (moderately severe impairment or worse).

c PR = 3.5 (95%CI 2.3 -5.3) among cases evaluated in sound-proof booth at hospital versus 2.6 (1.4 - 4.0) among cases evaluated at home.

d In three symptoms: vertigo, dizziness, unsteadiness.

**Figure 2:**
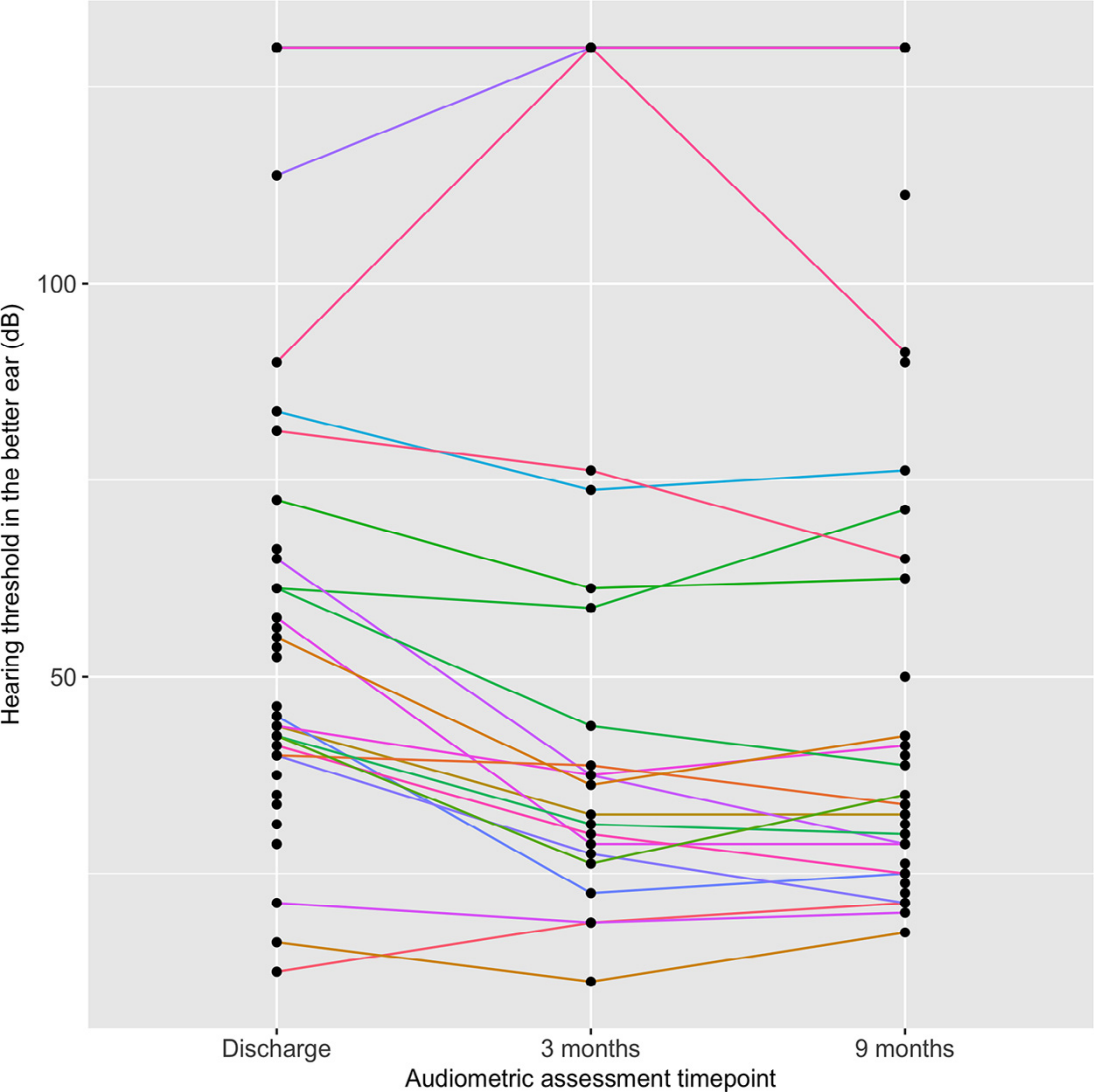
Change in hearing threshold over time in prospectively enrolled cases. Each line represents one case at 3 assessment time-points: at hospital discharge, 3 months and 9 months. Higher hearing threshold indicates a more severe level of hearing impairment. Cases with no response to sound stimuli at any time-point are grouped in the top line at a threshold >120 dB.

**Figure 3:**
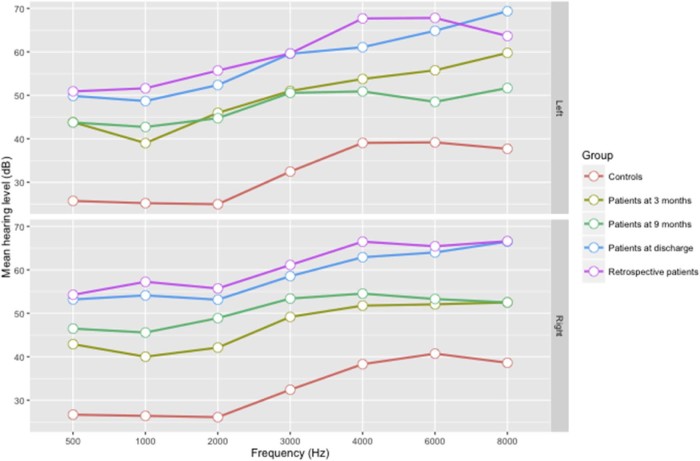
Mean hearing level among cases and controls who were responsive to sound stimuli for left and right ear across seven measured frequencies.

After discharge, hearing improved in 18/29 (62%) and 29/42 (69%), while deteriorated in 3/29 (10%) and 5/42 (12%) cases at 3 months and 9 months, respectively (Fig. 2). Most improvement was observed in non-severe hearing loss. Eight cases had no response to sound stimuli at all three assessments. Compared to discharge, the mean hearing thresholds were lower at 3 and 9 months for all frequencies (Fig. 3). The mean PTA was significantly lower at 3 months than at discharge (37.4 dB versus 46.3 dB, adjusted difference 11.1%, 95%CI7.0-15.1%) (Table 4). A similar difference was found at 9 months compared to discharge (36.4 dB versus 46.3 dB, adjusted difference 10.6%, 95%CI7.1-14.0%), indicating no significant improvement between 3 months and 9 months. Location of audiometry did not significantly affect the magnitude of change in hearing thresholds (Table 4).

**Table 4:** Analysis of change in hearing threshold averages among prospectively enrolled cases over time (between discharge and 3 months, and between discharge and 9 months) using linear mixed model controlling for between-patient variation

Model a	Assessment time-points b	Mean difference in PTA (95%CI)
Main effect with no adjustment	3 month follow-up	11.1% (7.0–15.1)
	9 month follow-up	10.6% (7.1–14.0)
Main effect with adjustment c	3 month follow-up	11.2% (7.0–15.1)
	9 month follow-up	10.5% (7.1–13.9)

PTA, Pure Tone Average; CI, Confidence Interval.

a Main effect was significant with p-value<0.001.

b Discharge time-point was the reference for comparison.

c Adjusted for age, sex, and location of audiometry (sound-proof booth at hospital versus home environment); the effect of these variables was non-significant. We also examined the effect of other potential confounding variables including corticosteroid treatment, preexisting medical conditions, alcohol drinking, occupation and education on hearing improvement, however no statistical significance was observed.

### Vestibular outcome

Balance disorder was observed in 37/44 (82%) of cases at discharge, and in 20/30 (67%) and 30/45 (65%) at 3 and 9 months, respectively (Table 2). Improvement was seen in 5/24 (21%) at 3 months and 8/35 (23%) at 9 months among those with balance disorders at discharge. However, there was only a slight decrease in the adjusted PRs from discharge to follow-up time-points among cases (Table 3). For balance disorder, the adjusted PRs are 2.8 (95%CI2.2-3.5) at discharge, 2.3 (1.6–3.0) at 3 months and 2.2 (1.6–2.9) at 9 months (Table 3). A similar ratio was found for the retrospectively enrolled cases (PR2.1, 95%CI1.4-2.8). Vestibular dysfunction, as determined by a failure of test condition 4, occurred in 16/32 (50%) of cases at 9 months (PR1.8, 95%CI1.1-2.5) and 13/24 (54%) of retrospectively enrolled cases (PR1.8, 95%CI1.1-2.6). All 18/45 cases (40%) who had severe-to-complete hearing loss at 9 months also had vestibular dysfunction.

Cases also reported more symptoms with a mean VSS score of 18.7 (95%CI12.0-25.5) at 3 months, 12.7 (8.6–16.7) at 9 months, and 22.1 (16.1–28.1) for retrospective cases, compared to 10.4 (9.1–11.7) in controls. Dizziness and unsteadiness, but not vertigo, were significantly more common in cases at 9 months and retrospectively enrolled cases (Table 3). All three symptoms were reported in 30% of cases at 3 months (PR5.8, 95%CI 2.6–12.8), 20% at 9 months (PR3.6, 1.5–8.2), and 29% of retrospectively enrolled cases (PR4.0, 1.8–8.9) compared to 4.4% in the controls. Among cases reporting at least one of the three symptoms at 9 months, 22/23 had balance disorder while 12/13 had vestibular dysfunction based on m-CTSIB findings.

### Other health outcomes

Proportion of cases experiencing problems on the five health domains is between 13–33% for cases at 3 months, 13–22% at 9 months and 3–32% for retrospectively enrolled cases (Table 2). Cases reported significantly more problems with mobility (at 9 months PR9.3), self-care (PR35.5), and performance of usual activities (PR42.5) (Table 3). Self-care problems were not significantly different among the retrospectively enrolled cases. No significant increase in pain and discomfort was observed for cases at 9 months and retrospectively enrolled cases, while a higher proportion for anxiety/depression was found in the controls. However, cases reported a significantly higher impact including emotional impact caused by hearing impairment and dizziness. No significant difference was observed in visual and cognitive performance.

## Discussion

Here we report the results of a case control study to prospectively examine the long-term sequelae of *S. suis* infection during 9 months post hospital discharge, and we evaluate the same outcomes in a group of retrospectively enrolled cases 11–34 months after discharge. We found significantly increased rates of hearing and vestibular impairments among cases. Profound/complete hearing loss (PTA ≥ 80 dB) was common among cases, which did not or hardly improve after 9 months. Likely, severe hearing loss post *S. suis* infection is irreversible given the similar rates between prospectively (at 9 months) and retrospectively (at 11–34 months) enrolled cases. Improvement within the first three months was observed among cases with non-severe hearing loss (<80 dB), consistent with current literature on hearing recovery after bacterial meningitis^17^ and other types of sensorineural hearing loss.^18^ Compared with results reported from a trial of dexamethasone vs. placebo for bacterial meningitis conducted in southern Viet Nam, rates of profound/complete hearing loss in our study were similar to the rates reported among *S. suis* patients in the placebo group (33.3% at discharge and 21.7% after 6 months).^4^ We conclude that non-severe hearing loss caused by *S. suis* infection may improve in the first three months post-discharge whereas severe hearing loss may not. Regardless of severity, the hearing status after 3 months is likely irreversible. We also explored the effect of corticosteroid treatment on vestibulocochlear function over time, but found no significant impact (see notes in Table 4 and data in Supplementary Material). It should be noted that this study was not powered to examine this effect.

We found that vestibular dysfunction occurs more often in cases with severe to complete hearing loss. This observation is in agreement with previous reports for different etiologies, that peripheral vestibular impairment was found mainly among those with residual deafness^19^ or co-incidence of balance system dysfunction was more frequent in profound hearing loss.^20^ Similar to hearing loss, vestibular imbalance persists in a large majority of cases after 9 months with a similar rate among retrospectively enrolled cases, suggesting a permanent loss of function. Future studies should investigate underlying mechanisms and find appropriate strategies to support the recovery process of vestibular function in these patients.

To measure vestibular outcome, we used the postural metric m-CTSIB, which has been reported to have 90% sensitivity and 95% specificity in patients with vestibular disorders^21^ and shown non-inferior compared to computerized dynamic posturography instruments in clinical settings.^22^ Using a similar test, the reported prevalence among adults aged 40 years and older was 35.4% in a national survey in the United States.^12^ Rates of self-reported vertigo in community samples in the United Kingdom and Finland were 21–29%.^23^, ^24^, ^25^ The rates of vestibular dysfunction and symptoms in our controls were comparable to these community-based rates. Our estimates for *S. suis* patients were in the high range of the reported rates in previous studies of *S. suis* infection (3–60%).^2^ However, we also found similarly high rates of self-reported vertigo, dizziness and unsteadiness, furthering supporting the validity of our estimates using the m-CTSIB.

Survivors of *S. suis* infection have a significantly lower health status than controls. The prevalence of experiencing problems across the five EQ-5D domains among patients in this study (aged between 28–79 years) are comparable to a sample from the general population aged 60 years and older in a rural setting in Viet Nam (10–39%).^26^ The higher rate of anxiety/depression among our controls was expected since emotional distress is one of the problems that visitors are faced with while providing care to patients. Nonetheless, cases scored worse for emotional and social domains than controls on the hearing and dizziness specific scales.

One limitation of this study was that age matching was not fully achieved, as a result we did not reach the ratio of 1:4 for cases and controls. Therefore we included all recruited controls in the analyses for both case groups, and age was included as a potential confounder in our analyses. Secondly, 9-month audiometry was done at the home environment for some patients, which may have led to an overestimation of hearing loss prevalence and severity among cases. Nonetheless, cases evaluated at home had a smaller PR than those evaluated in the sound-proof booth as shown in the analysis stratified by location of audiometry at 9 months, despite having overlapping CIs (see notes in Table 3). Thirdly, controls were all caretakers of hospital admitted family members and therefore may have experienced a disproportionate amount of anxiety/depression than if we had recruited controls from the general population. Finally, we did not formally measure tinnitus. Tinnitus and hearing loss are often correlated, although the rate of tinnitus among cases with hearing loss varies greatly.^27^ In studies among noise-exposed workers, tinnitus increased with more severe hearing loss, ranging from 5–10% in mild to 12–30% in profound-to-complete loss.^28^ The co-incidence of tinnitus could amplify the impact of hearing loss on patients. Among the retrospectively enrolled patients we contacted, tinnitus was reported in 3/21 of those who reported hearing impairment, illustrating this may be common and lower the quality of life.

In conclusion, this study is the first to provide strong evidence that a large proportion of patients who survive *S. suis* infection suffer from serious hearing and vestibular impairment. Hearing mostly improves in the first 3 months after discharge among the less severely affected cases, while vestibular dysfunction shows little recovery. Patients also experience multiple symptoms, which aggravate the impact of disease sequelae on their health and quality of life. Further research is required to identify appropriate treatment strategies to reduce the incidence and severity of hearing and vestibular impairment in survivors of *S. suis* infection.

## Conflicts of interest

All authors declare that they have no conflicts of interest in relation to the present manuscript.

## Role of the funding source

The funder had no role in the design, collection, analysis, interpretation of the data, writing the report, or in the decision to submit the manuscript.

## Footnotes

a Reproduced by special permission of the Publisher, Psychological Assessment Resources, Inc., 16204 North Florida Avenue, Lutz, Florida 33549, from the Mini Mental State Examination, by Marshal Folstein and Susan Folstein, Copyright 1975, 1998, 2001 by Mini Mental LLC, Inc. Published 2001 by Psychological Assessment Resources, Inc. Further reproduction is prohibited without permission of PAR, Inc. The MMSE can be purchased from PAR, Inc. by calling (800) 331–8378 or (813) 968–3003.
