# Supplementary material for: Long-term outcomes of patients with Streptococcus suis infection in Viet Nam: A case-control study
Source: J Infect. 2018 Feb;76(2):159–67. doi: 10.1016/j.jinf.2017.09.019 (PMC5790056; doi:10.1016/j.jinf.2017.09.019)
Supplement: Appendix S1 — Supplementary materials. [file mmc1.docx]

**Title:** Long-term outcomes of patients with *Streptococcus suis* infection in Viet Nam: a case-control study

**Author names:** Vu T.L. Huong , Hoang B. Long, Nguyen V. Kinh, Ta T.D. Ngan, Vu T.V. Dung, Behzad Nadjm, H. Rogier van Doorn, Ngo T. Hoa, Peter Horby, Heiman F.L. Wertheim

**Supplementary Material**

**Methods**

All patients and controls were assessed for hearing levels, balance, visual acuity, cognitive performance, history of vestibular symptoms, impact of hearing impairment and dizziness, and overall health status as in the following table.

Supplementary Table 1. Study assessments and instruments for patients and controls

| Type of participant | Clinical Exam. | Audiometry, Balance, Visual acuity test | MMSE | Questionnaire (HHIA, VSS, DHI, EQ-5D) |
| --- | --- | --- | --- | --- |
| Prospective patient |  |  |  |  |
| Discharge | X | X | X |  |
| 3 months follow-up | X | X | X | X |
| 9 months follow-up | X | X | X | X |
| Retrospective patient | X | X | X | X |
| Control |  | X | X | X |

MMSE: Mini Mental State Examination; HHIA: Hearing Handicap Inventory for Adults; VSS: Vertigo Symptoms Scale; DHI: Dizziness Handicap Inventory, EQ-5D: a short questionnaire on generic health status.

*Clinical examination*

This includes an evaluation of mental status, motor function and balance, sensory examination, reflexes, cranial nerve evaluation (for patients only). Study doctors performed clinical examination on patients at hospital discharge (prospective patients) and at follow-up visits (all patients).

*Audiometry*

The hearing threshold in each ear was tested for frequencies from 0.5 to 8kHz with the first threshold tested at 1kHz. For quality control, a second measure at 1kHz was included to check if the discrepancy between two threshold measurements was within 10 decibels. All hearing thresholds are reported in dB HL (American National Standards Institute, 2004).

*The Modified Clinical Test of Sensory Interaction and Balance (m-CTSIB) [1]*

This test was designed to test the participant’s ability to stand unassisted with feet together (closed stance) and arms folded across the waist with both hands holding the elbows, under 4 successive test conditions: eyes open on firm surface, eyes closed on firm surface, eyes open on foam surface and eyes closed on foam surface (Table below). A Sunmate medium density foam pad (40cm x 45cm x 8cm) was used in test condition 3 and 4. The test was scored on a pass/fail basis. It was scored as fail if the subject moved their feet or hands off the starting position, began to fall, or required assistance to maintain balance, or opened their eyes in the conditions that required eyes closed. If the subject failed under any condition, a second attempt was permitted (with a maximum of 2 attempts allowed under each condition). The test was discontinued whenever he/she failed to pass a condition on both attempts.

Supplementary Table 2. Four test conditions of the Modified Clinical Test of Sensory Interaction and Balance (m-CTSIB) test used for *S. suis* patients and controls

| Test condition | Description | Sensory input |
| --- | --- | --- |
| Condition 1 | Eyes open, stand on firm surface, 15 seconds | Visual, proprioceptive, vestibular system |
| Condition 2 | Eyes closed, stand on firm surface, 15 seconds | Proprioceptive, vestibular system |
| Condition 3 | Eyes open, stand on foam surface, 30 seconds | Visual, vestibular system |
| Condition 4 | Eyes closed, stand on foam surface, 30 seconds | Vestibular system |

These conditions examine the function of three sensory inputs contributing to balance including the vestibular system, vision, and proprioception. Balance disorder was determined as a failure to complete the test at any condition, while vestibular dysfunction was determined as a failure to pass the test condition 4 only (excluding those who did not pass the previous 3 conditions and thus did not participate in condition 4). These conditions examine the function of three sensory inputs contributing to balance including the vestibular system, vision, and proprioception. This testing protocol has been used in an on-going cross-sectional national health and nutrition examination survey in the U.S [2]. Previous studies have shown that inability to remain standing on the foam with eyes closed indicate a vestibular dysfunction with 90% sensitivity and 95% specificity [3].

*Visual acuity test*

Visual acuity was tested using a locally available Snellen chart (letter C) at 5-meter distance. Vision impairment is defined in two categories as used by the Vision Loss Expert Group in the Global Burden of Disease study [4]:

- Moderate and severe vision impairment: defined as visual acuity in the better eye < 6/18 but ≥ 3/60 at presentation
- Blindness: defined as visual acuity in the better eye < 3/60 at presentation

If the subject is wearing correction at presentation, visual acuity while wearing correction is used.

*Mental status*

Mental status is assessed using the Mini Mental State Examination (MMSE) [5] on various areas of cognitive function including orientation, registration, attention, calculation, recall, and language^(Footnote^[[1]](#footnote-1)^)^ It has been widely used for assessing cognitive mental status in both clinical and research settings because of its brevity and ease in administration [6]. Maximum score is 30. A score of 24-30 is considered as no cognitive impairment, 18-23 mild cognitive impairment, and below 18 severe cognitive impairment [7].

*Hearing Handicap Inventory for Adults (HHIA)*

We used the 25-item Hearing Handicap Inventory for Adults (HHIA) [8] questionnaire to assess the emotional (13 items) and social/ situational (12 items) problems associated with hearing loss. For each item, a score of 0, 2 or 4 is assigned to the corresponding response as “no”, “sometimes” or “yes”, making the total score ranging from 0 to 100. Total HHIA scores are polytomized into three categories:

- No handicap: score between 0-16
- Mild-Moderate handicap: 18-42
- Significant handicap: 44+

*Dizziness Handicap Inventory (DHI)*

Dizziness Handicap Inventory (DHI) [9] was used to assess the perceived handicap caused by vestibular dysfunction. Three domains were examined: functional (9 items about the impact of dizziness on their daily activities), emotional (9 items about the impact of dizziness on the person’s emotional wellbeing), and physical (7 items about the impact of physical activities on the person’s sense of instability). For each item, a score of 0, 2 or 4 is assigned to the corresponding response as “no”, “sometimes” or “yes”, making the total score ranging from 0 to 100. Following categories are analyzed:

- No handicap: score between 0-14
- Mild handicap: 16-34
- Significant handicap: 36+

*Baseline characteristics*

Baseline characteristics include sex, age (years), location (rural versus urban), education (years of schooling), occupation (farming/ manual work versus others), and pre-existing medical conditions. Pre-existing conditions include one or more of the following: arthritis, diabetes, hepatitis/ cirrhosis, renal disease, hypertension, heart disease, chronic lung disease, seizure, brain injury, paralysis, splenectomy, currently on steroids treatment, depression, HIV, malignancy, or any other immunosuppressed conditions. Corticosteroid treatment during hospital admission for patients was also obtained based on medical records. These baseline characteristics are compared between patients and controls and included in the main analyses for outcomes.

**Results**

Supplementary Table 3. Mean difference in hearing level at each frequency

| Frequency  (Hz) | Left ear | | Right ear | |
| --- | --- | --- | --- | --- |
|  | 3 months | 9 months | 3 months | 9 months |
| 500 | 11.1 (5.3-6.6) | 10.0 (5.1-14.6) | 10.0 (3.9-15.8) | 9.6 (4.6-14.4) |
| 1000 | 10.9 (4.7-16.6) | 13.8 (8.8-18.4) | 10.6 (5.2-15.6) | 10.9 (6.6-15.1) |
| 2000 | 15.0 (9.3-20.3) | 16.0 (11.3-20.4) | 12.8 (5.1-19.8) | 13.0 (6.7-18.8) |
| 3000 | 11.1 (6.3-15.6) | 11.9 (8.0-15.6) | 11.1 (2.9-18.6) | 12.1 (5.5-18.2) |
| 4000 | 9.6 (1.8-16.7) | 8.8 (2.3-14.9) | 8.5 (2.7-13.8) | 9.1 (4.5-13.5) |
| 6000 | 10.1 (2.6-17.0) | 14.2 (8.2-19.8) | 10.0 (2.2-17.2) | 12.5 (6.2-18.3) |
| 8000 | 9.8 (0.8-17.9) | 14.6 (7.5-21.1) | 11.8 (3.4-19.6) | 12.6 (5.7-18.9) |

Data are presented as percentage (%) of change compared to the corresponding value at discharge, adjusted for age and sex.

Supplementary Table 4. Hearing outcome among patients with and without corticosterioid treatment during hospitalization

| Group | Prospective cases, n(%) | | | | | | | | | Retrospective cases, n(%) (n=31) | | |
| --- | --- | --- | --- | --- | --- | --- | --- | --- | --- | --- | --- | --- |
|  | Discharge  (n=45) | | | 3 months  (n=30) | | | 9 months  (n=45) | | |  |  |  |
| PTA (dB) | <34 | 35-80 | >80 | <34 | 35-80 | >80 | <34 | 35-80 | >80 | <34 | 35-80 | >80 |
| Corticosterioid treatment | 4  (14) | 16  (57) | 8  (29) | 7  (37) | 6  (32) | 6  (32) | 11  (38) | 11  (38) | 7  (24) | 9  (53) | 3  (18) | 5  (29) |
| Without  corticosterioid  treatment | 3  (38) | 7  (38) | 6  (24) | 3  (27) | 3  (27) | 5  (46) | 8  (50) | 2  (12) | 6  (38) | 2  (14) | 9  (64) | 3  (22) |

Data are the number (%) of patients in each group. PTA: Pure Tone Average

**References:**

1. Wrisley DM, Whitney SL. The effect of foot position on the modified clinical test of sensory interaction and balance. Arch Phys Med Rehabil **2004**; 85: 335–8.
2. Agrawal Y, Carey JP, Hoffman HJ, Sklare DA, Schubert MC. The modified Romberg Balance Test: normative data in U.S. adults. Otol Neurotol **2011**; 32: 1309–11.
3. Maranhão-Filho PA, Maranhão ET, Silva MMd, Lima MA. Rethinking the neurological examination I: static balance assessment. Arq Neuropsiquiatr **2011**; 69: 954–8.
4. Bourne R, Price H, Taylor H, Leasher J, Keeffe J, Glanville J, et al. New systematic review methodology for visual impairment and blindness for the 2010 Global Burden of Disease study. Ophthalmic Epidemiol **2013**; 20: 33–9.
5. Folstein MF, Folstein SE, McHugh PR. "Mini-mental state". A practical method for grading the cognitive state of patients for the clinician. J Psychiatr Res **1975**; 12: 189–98.
6. Guerrero-Berroa E, Luo X, Schmeidler J, et al. The MMSE orientation for time domain is a strong predictorof subsequent cognitive decline in the elderly. Int J Geriatr Psychiatry **2009**; 24: 1429–37.
7. Tombaugh TN, McIntyre NJ. The mini-mental state examination: a comprehensive review. J Am Geriatr Soc **1992**; 40: 922–35.
8. Newman CW, Weinstein BE, Jacobson GP, Hug GA. Test-retest reliability of the hearing handicap inventory for adults. Ear Hear **1991**; 12: 355–7.
9. Jacobson GP, Newman CW, Hunter L, Balzer GK. Balance function test correlates of the Dizziness Handicap Inventory. J Am Acad Audiol **1991**; 2: 253–60.

1. - ^Footnote^ Reproduced by special permission of the Publisher, Psychological Assessment Resources, Inc., 16204 North Florida Avenue, Lutz, Florida 33549, from the Mini Mental State Examination, by Marshal Folstein and Susan Folstein, Copyright 1975, 1998, 2001 by Mini Mental LLC, Inc. Published 2001 by Psychological Assessment Resources, Inc. Further reproduction is prohibited without permission of PAR, Inc. The MMSE can be purchased from PAR, Inc. by calling (800) 331-8378 or (813) 968-3003.

   [↑](#footnote-ref-1)
